# Effectiveness of Pediatric Teleconsultation to Prevent Skin Conditions in Infants and Reduce Parenting Stress in Mothers: Randomized Controlled Trial

**DOI:** 10.2196/27615

**Published:** 2022-02-17

**Authors:** Tomohisa Ando, Rintaro Mori, Kenji Takehara, Mari Asukata, Shuichi Ito, Akira Oka

**Affiliations:** 1 Department of Pediatrics University of Tokyo Tokyo Japan; 2 Department of Health Policy National Center for Child Health and Development Tokyo Japan; 3 Population Ageing and Sustainable Development Asia and the Pacific Regional Office United Nations Population Fund Bangkok Thailand; 4 Tsurumi Ward Administration Office Yokohama Japan; 5 Department of Pediatrics Graduate School of Medicine Yokohama City University Yokohama Japan

**Keywords:** teleconsultation, email newsletters, eHealth, pediatrician, infant, mother, atopic dermatitis, parenting stress, randomized controlled trial, stress, dermatitis, parenting, pediatrics, email, children, Japan

## Abstract

**Background:**

In Japan, eHealth for infants and mothers is currently an important aspect, but its effectiveness has rarely been examined. For infants, skin problems, including atopic dermatitis (AD), which is known to lead to other allergic diseases, are one of the most common conditions. Mothers of infants are prone to experiencing parenting stress, which adversely affects mothers’ and children’s well-being. Additionally, studies have reported that AD among offspring enhances parenting stress, and postnatal maternal psychological problems can increase the risk of AD in children.

**Objective:**

This study evaluated the effectiveness of pediatric teleconsultation for preventing AD in infants and reducing parenting stress in mothers in Japan.

**Methods:**

The study was an open-label, randomized, parallel-armed controlled trial. In total, 318 pairs of infants and mothers in the Yokohama City Sakae Ward were recruited when they submitted birth cards to the ward, received the explanation about the trial, and provided informed consent on the website for this trial. Eligible pairs of infants and mothers were randomly assigned to the intervention group (n=140) or the control group (n=138). Participants in the intervention and control groups received routine postnatal care from local government services. In addition, participants in the intervention group had the option to combine routine pediatric services with teleconsultation and email newsletters without charge from the date of registration until the infant turned 4 months. Primary outcomes were (1) the prevalence of AD in infants diagnosed based on the United Kingdom Working Party criteria and (2) parenting stress and mental status of mothers assessed using the Parenting Stress Index-Short Form (PSI-SF) and General Health Questionnaire-12 (GHQ-12). Data were collected by the ward office staff and researcher during the 4-month checkup.

**Results:**

The prevalence of AD in infants was significantly lower in the intervention group than in the control group during the 4-month checkup (20% vs 33%, *P*=.02; relative risk ratio, 0.709 [95% CI 0.519-0.969]). No significant differences were observed in the PSI-SF and GHQ-12 scores between the 2 groups. There was a significant difference in the prevalence of AD between participants who used teleconsultation services and email newsletters, participants who did not use teleconsultation services but received email newsletters, and participants who neither received nor used either service (18% vs 22% vs 33%, *P*=.048).

**Conclusions:**

This is the first randomized controlled trial demonstrating that routine pediatric care combined with teleconsultation and email newsletters was effective in reducing the prevalence of AD in infants. The findings highlight the potential of pediatric eHealth to become a useful new strategy for preventing AD.

**Trial Registration:**

University Hospital Medical Information Network Clinical Trials Registry UMIN000029774; https://upload.umin.ac.jp/cgi-open-bin/ctr_e/ctr_view.cgi?recptno=R000034022

## Introduction

The exchange of valid health-related information between health care professionals and their patients can facilitate successful diagnosis, treatment, and prevention as well as research and evaluation, thereby advancing the health of individuals and communities. In this context, telemedicine and eHealth refer to the location-independent delivery of health care services through information and communication technologies. Telemedicine and eHealth are widely used services due to their functionality, ease of use, cost-effectiveness, time-saving features, and accessibility for patients who live far from health care facilities [1-3]. eHealth for expectant or nursing mothers has attracted particular attention because women of reproductive age are particularly frequent users of the internet, social media, and smartphone apps and thus appear to be prime candidates for this form of health care delivery [4].

Telemedicine and eHealth intervention services provided to pediatric patients with conditions such as autism, attention deficit hyperactivity disorder, and chronic diseases have been demonstrated to positively influence pediatric patients as well as families and health care providers [5-8]. Systematic reviews have indicated that telemedicine services for pediatric care result in comparable or improved outcomes relative to in-person services [7,8]. Moreover, parents have reported experiencing actual and perceived benefits of eHealth services, including ease of use, higher confidence in and satisfaction with infant care, and efficient knowledge transmission [9].

In Japan, a few reports have described the use of perinatal eHealth services provided by health care providers, such as telediagnosis for fetal cardiac diseases [10-12], construction of electronic medical records for perinatal care [13], and digitalization of a maternal and child health handbook [14]. Moreover, no randomized controlled trial (RCT) has examined the effectiveness of eHealth for infants and mothers.

As one of the most common conditions afflicting newborns and young infants, skin disorders appear to be prime candidates for telemedicine services. More than 50% of newborns and young infants have skin conditions such as infantile eczema, diaper dermatitis, intertrigo, and seborrheic dermatitis [15]. In particular, atopic dermatitis (AD) or skin inflammation should be prevented because it may increase the risk of other allergic diseases [16]. Previous RCTs on eHealth for AD have focused on patients [17,18], the feasibility of web-based consultations [19], or equivalence to ordinary in-person care [20]. However, to date, no study has examined whether eHealth can prevent infants from developing AD.

Parents of children with AD encounter significant and persistent challenges to successfully manage the condition [21]. Mothers, who are often the primary caregivers, experience a higher degree of psychological stress while raising offspring with serious illnesses, with corresponding negative impacts on parents’ and children’s well-being [22]. A mother’s mental state—manifested in low maternal sensitivity, perinatal anxiety, or postpartum depression—is a modifiable risk factor for the development of childhood AD [23-25]. Recent studies have emphasized the need for and usefulness of parenting training programs as easily accessible interventions to help reduce parental stress [26,27]. Thus, adequate psychological support and training would help in reducing parental stress and in better management of the condition of atopic children.

In Japan, reports from the Sukoyaka (meaning “healthy and happy” in Japanese) Family 21, a national campaign for maternal and child health, have highlighted the ineffectiveness of current measures to improve women’s mental health, the insufficiency of childcare and development consultation services, and the necessity of support from pediatricians as well as obstetricians, midwives, and public nurses [28,29]. Studies conducted in other countries indicate that the use of eHealth-based services in such scenarios can effectively and feasibly address problems among parents of children with AD [30-33]. Accordingly, we propose that telemedicine services could reduce parental vulnerability by addressing knowledge deficits regarding disease management and parenting methods while also alleviating stress-inducing logistical barriers such as travel, time, and cost.

In this study, we implemented pediatric teleconsultation and evaluated its effectiveness for improving skin conditions in infants, especially in terms of preventing AD and reducing parenting stress in mothers in Japan. Compared with the control group receiving routine postnatal care via local government services, we hypothesized that the additional service comprising teleconsultation and email newsletters provided by pediatricians would be an effective strategy for preventing AD in infants and reducing parenting stress in mothers at 4 months postpartum.

## Methods

### Study Design

This study was an open-label, randomized, parallel-armed, controlled trial. Data collection was blinded to the intervention assignment of the participants. We registered this trial design, including the hypotheses and outcome measures, at the University Hospital Medical Information Network Clinical Trials Registry (trial registration number: UMIN000029774), although we did not publish the protocol paper of this study.

### Participants, Recruitment, and Randomization

The study participants were infant and mother dyads in the Yokohama City Sakae Ward. The pairs were recruited when mothers submitted their infants’ birth cards (a document submitted to the local government upon an infant’s birth) at the Yokohama City Sakae Ward Office, received an explanation about the study, and provided informed consent on the Sakae Infants and Mothers with the Pediatric Teleconsultation (SIMPLE) Study website that was exclusively set up for this study. Recruitment took place from November 1, 2017, to May 31, 2018. There were no other specific inclusion criteria for the trial.

Exclusion criteria included the unwillingness or inability to comply with the study requirements and procedures; a discretionary decision by study staff that either the mother or the infant might not be suitable for participating in this trial; a mother’s inability to understand Japanese (although communication through a Japanese interpreter could be performed in exceptional circumstances); infants born before 37 weeks of gestation; infants born after 42 weeks of gestation; twins or multiple births; and infants needing neonatal intensive care. These criteria were available for review on the SIMPLE Study website. We did not exclude any infant-mother pairs following the development of maternal mental health problems or infant skin problems.

Shortly after enrollment, a simple randomization method was used to assign participants to the intervention or control group with a ratio of 1:1 in the order of registration on the SIMPLE Study website.

### Intervention

In addition to routine postnatal care through local government services such as home visits by public nurses and breastfeeding consultation services by midwives, participants in the intervention group had the option to use a pediatric teleconsultation service named “Syounika (meaning “pediatric” in Japanese) Online” and receive email newsletters without any charge from the time of registration to 4 months of age. The email newsletters were delivered once every 3 days (30 times in total) during the intervention period.

**Figure 1:**
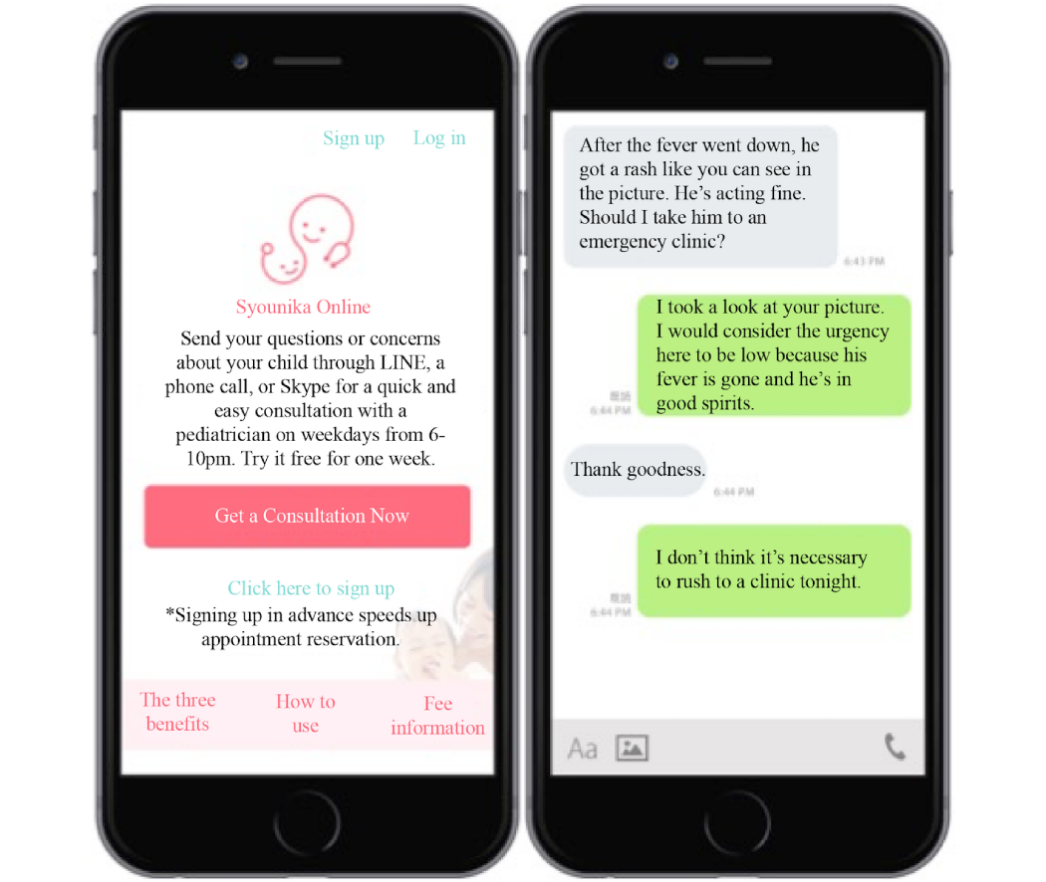
Syounika Online Service (translated from Japanese to English). Skype was no longer available during the trial.

The Syounika Online Service was launched by Kids Public Inc in 2016. The core service is teleconsultation through video calls, voice calls, or text messages via smartphones. Users can consult with pediatricians about any matters regarding their children’s health and parenting for 10 minutes from 6 PM to 10 PM on weekdays (Figure 1). Syounika Online also delivers email newsletters containing useful information about children’s health and parenting, such as infant skin care, breastfeeding, and maternal self-care. Syounika Online collaborated with local governments, companies, health insurance providers, and paid subscribers to recruit users across the country.

**Figure 2:**
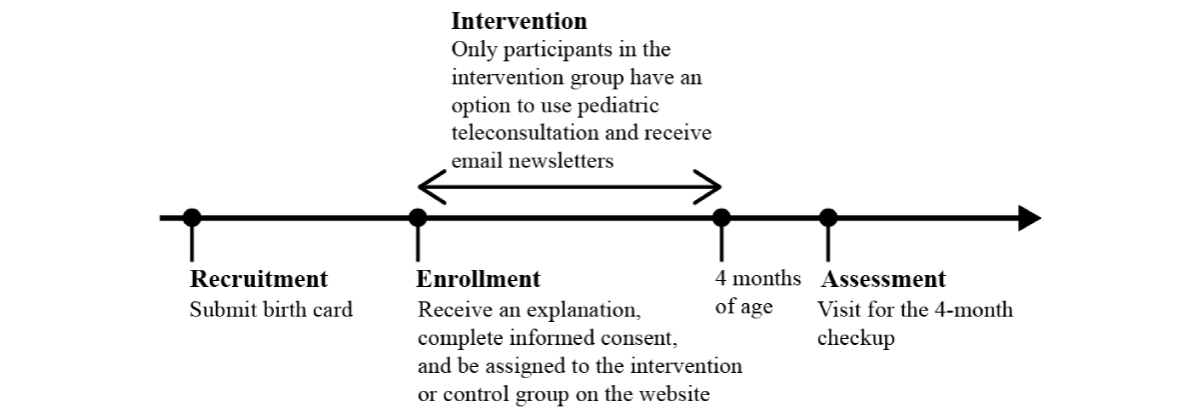
Outline of the study flow.

All data were collected by the ward office staff and the researcher at the community health center in the Yokohama City Sakae Ward when participants visited there for the 4-month checkup. Figure 2 describes the flow of the study.

### Primary Outcomes

The primary outcomes were the prevalence of AD in infants, parenting stress, and mothers’ mental health status at the 4-month checkup.

#### Prevalence of AD in Infants

The diagnosis of AD was based on the United Kingdom Working Party (UKWP) criteria [34]. In addition to itchy skin in the last 12 months, the infant should meet at least three of the following criteria: (1) history of skin creases, such as in the folds of the elbows, behind the knees, fronts of the ankles, or around the neck (including the cheeks in children under 10 years); (2) a personal history of asthma or hay fever (or history of atopic disease in a first-degree relative for children under 4 years); (3) a history of generally dry skin in the last year; (4) visible flexural eczema (or eczema involving the cheeks or forehead and the outer limbs in children under 4 years); (5) onset under the age of 2 years (not applicable if child is currently under 4 years). All the infants were assessed by 1 pediatrician who was blinded to the intervention assignment of participants to ascertain whether there was visible flexural eczema.

#### Parenting Stress and Mental Status of Mothers

Parenting stress was measured using the original Japanese version of the Parenting Stress Index-Short Form (PSI-SF; 19 items)—an abridged version of the full-length PSI [35]—which was developed in 1983 for screening and triage purposes to evaluate the parenting system and identify issues that may cause problems in the child's or parent's behavior. The Japanese version of the PSI-SF has been demonstrated as a valid and reliable tool, and it consists of 19 items and 2 subscales, namely parental stress and child-related stress [36,37]. Higher PSI-SF scores indicated higher levels of parental stress. Araki et al reported a Cronbach alpha coefficient of .82 for the total PSI-SF score [36]. The Cronbach alpha coefficient in this study was .873.

Current mental health was measured using the General Health Questionnaire-12 (GHQ-12; 12 items) [38]. GHQ-12 is a valid, internationally used measure of mental status in a population and has been used in several studies to assess the medium- and long-term postpartum mental health status of mothers [39]. Participants with GHQ-12 scores ≥4 were classified as the high GHQ score group [40]. Higher GHQ-12 scores indicated higher severity of a mental health problem. The Cronbach alpha coefficient in this study was .774.

### Secondary Outcomes

Secondary outcomes were breastfeeding rates and the developmental quotient (DQ) of infants, which were examined by a pediatrician through questionnaires at the 4-month checkup. Breastfeeding rates were determined by asking mothers through a questionnaire about the proportion of time spent on breastfeeding the infants (100%, 80%-99%, 20%-79%, 1%-19%, or 0%). The DQ was assessed using the Enjoji Scales of Infant Analytical Development, which has been universally used in Japan [41]. This test can be administered to infants and children ranging from newborns to 4 years and 7 months, and it assesses 6 categories (locomotor, manual activity, language, emotional status, intelligence, and social behavior). The DQ was calculated using the following formula: DQ = (assessed age / real age) × 100.

### Sample Size

Because no previous studies have examined the effectiveness of eHealth in this setting, we estimated the required sample size from the limited data on the prevalence of AD and the PSI-SF total scores presented in previous studies [37,42].

Based on the prevalence of AD at the 4-month checkup, we estimated a sample size of 132 infants per group, with an AD prevalence of 18% in the intervention group and 33% in the control group, α=.05, and a power of 80% ([1– β] × 100, where β=.2).

Based on the PSI-SF total scores at the 4-month checkup, we estimated a sample size of 86 mothers per group with the mean and SD of the PSI-SF total scores being 37 and 7, respectively, in the intervention group and 40 and 7, respectively, in the control group, along with α=.05 and β=.2. These values were approximated by referring to the PSI parenting stress index handbook [37].

Overall, we expected a dropout rate of approximately 10% and set the target number of participants to 300, which was estimated as follows: There were an estimated 132 infants per group, thus making 264 dyads. Assuming a 10% nonresponse rate, 264 would correspond to 90% of the study participants. To factor in nonresponses, the sample size was inflated by a factor of 0.9, that is 293 (264/0.9), which was rounded up to 300. This sample size was insufficient for detailed analysis of GHQ-12 score groups, but we expect our data to contribute to future meta-analyses on this topic.

### Statistical Analysis

Data were analyzed by intention-to-treat using SPSS version 25.0 (IBM Corporation). For categorical and count characteristic variables (AD, GHQ-12≥4, and breastfeeding rate), we used the Pearson chi-square or Fisher exact test to detect statistical differences in the distributions between the intervention and control groups. For continuous characteristic variables (PSI-SF total stress, child domain, parent domain, locomotion, hand exercise, common practice, human interaction, speech, language comprehension, and average of DQ), we tested for differences between the 2 groups using the *t* test for normally distributed variables and the Mann-Whitney *U* test for non-normally distributed variables. The results of the outcomes assessed at the 4-month checkups were analyzed using a per-protocol analysis to examine the individual effectiveness of the teleconsultation and email newsletters by comparing three groups: participants who received and used teleconsultation services and email newsletters; participants who did not use teleconsultation services but received email newsletters; and participants who neither received nor used either service.

In presenting the differences between the groups, we calculated the differences as follows: For categorical variables, we calculated differences in percentages; for continuous variables, we calculated differences between means. We set the level of statistical significance at 0.05.

### Ethical Considerations

This study received ethical approval from the institutional review board of the National Center for Child Health and Development before participant recruitment commenced (approval number: 1570). We obtained informed consent on the internet from the adult participants at the time of enrollment. The protocol for this study is available on the SIMPLE Study website. We arranged for participants in the control group to have access to Syounika Online without charge from 5 to 8 months of age. We did not prohibit any participants from using Syounika Online at their own expense.

## Results

### Participant Details

**Figure 3:**
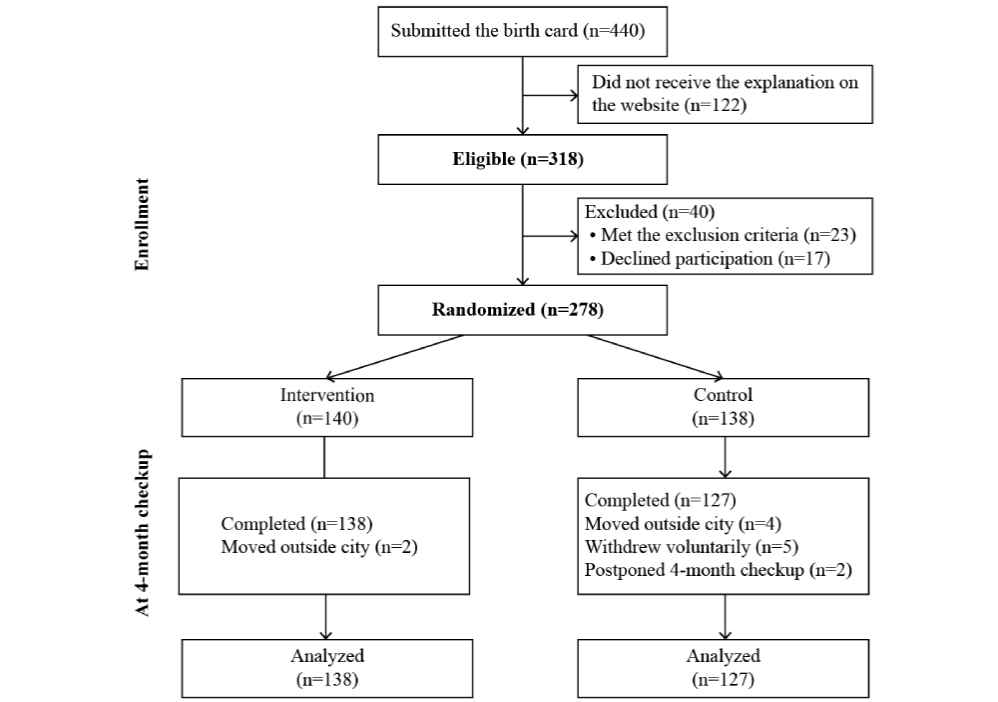
Participant flowchart.

Among the 440 pairs of infants and mothers who submitted their birth cards to the Yokohama City Sakae Ward Office during the recruitment period, we assessed the eligibility of the 318 pairs who accessed the SIMPLE Study website to receive an explanation of this study (Figure 3). In total, 23 pairs were excluded because infants were born before 37 weeks or after 42 weeks of gestation, and there were twins or multiple births, or infants requiring neonatal intensive care. Further, 17 pairs declined participation. Finally, 278 infant-mother pairs were enrolled. We allocated 140 pairs to the intervention group and 138 pairs to the control group. Furthermore, 13 pairs were excluded from the analysis because 6 moved out of the city, 5 withdrew participation voluntarily, and 2 postponed their 4-month checkups. No pairs crossed over between trial arms. Finally, we analyzed the data of 265 infant-mother dyads among the 278 pairs (95.3%).

Table 1 summarizes the characteristics of the participants and their families. We detected no significant differences in their characteristics.

**Table 1:** Characteristics of infants and mothers in intervention and control groups (N=265).

Characteristics			Intervention					Control					*P* value^a^
			Values		n^b^		Values			n			
**Infants**													
	Female, n (%)	67 (48.6)		138		52 (40.9)			127		.22		
	Birth weight (g), mean (SD)	3102 (359.8)		138		3060 (391.8)			127		.45		
	Birth height (cm), mean (SD)	49.4 (1.8)		138		49.3 (1.9)			127		.75		
	Neonatal problem, n (%)	18 (13.1)		137		21 (16.5)			127		.49		
**Mothers**													
	Age (years), mean (SD)	33.0 (4.8)		138		33.0 (5.0)			127		.90		
	Employment, n (%)	68 (50.4)		135		61 (49.2)			124		.90		
	History of hospitalization or long-term treatment, n (%)	30 (21.7)		138		28 (22.1)			127		.99		
	Current illness, n (%)	13 (9.5)		137		15 (11.8)			127		.56		
	Parity: primiparous, n (%)	67 (48.6)		138		63 (49.6)			127		.90		
	Gravidity, mean (SD)	1.9 (1.0)		137		2.1 (1.5)			127		.62		
	Threatened abortion or premature delivery, n (%)	19 (14.0)		136		11 (8.7)			127		.24		
	Gestational age (weeks) , mean (SD)	39.0 (1.1)		138		39.0 (1.1)			127		.88		
**Method of birth, n (%)**												.99	
	Spontaneous vaginal birth	102 (73.9)		138		94 (74.0)			127				
	Instrumental vaginal birth	14 (10.1)		138		13 (10.2)			127				
	Cesarean section	22 (15.9)		138		20 (15.8)			127				
**Delivery location, n (%)**												.85	
	Hospital	70 (50.7)		138		64 (50.8)			126				
	Clinic	68 (49.3)		138		61 (48.4)			126				
	Maternity home	0 (0.0)		138		1 (0.8)			126				
**Families**													
	Father’s age (years), mean (SD)	34.6 (6.2)		138		34.6 (5.8)			127		.98		
	Father’s employment, n (%)	132 (97.8)		135		126 (99.2)			127		.62		
	Family members who smoke, n (%)	39 (28.3)		138		36 (28.4)			127		.99		
	Family history of atopic dermatitis, n (%)	32 (23.2)		138		40 (31.5)			127		.17		
	Family history of bronchial asthma, n (%)	38 (27.7)		137		30 (23.6)			127		.48		

^a^*P* values for categorical variables were calculated using either the Pearson chi- square or Fisher exact test; *P* values for continuous variables were calculated using *t* tests.

^b^Some n values are lower than others due to missing data.

### Usage Status of Syounika Online

Among the 138 intervention group participants, 59 (42.4%) used the teleconsultation service 116 times. Skin problems in infants (n=32, 28%) were the most common reasons for consultations, followed by gastrointestinal problems (n=26, 22%) and breastfeeding problems (n=15, 13%). In the control group, 1 participant used the teleconsultation service 17 times at her own expense.

The email newsletters from Syounika Online were delivered 30 times and contained 28 pieces of advice on skin care in infants and 25 pieces of advice on breastfeeding. Overall, the 138 participants in the intervention group and 1 participant in the control group received the newsletters, and their opening rates were 30% to 58%.

### Main Analysis

The results of the primary and secondary outcomes are summarized in Table 2.

Analysis of the primary outcomes revealed that the prevalence of AD was significantly lower in the intervention group than in the control group (20% vs 33%, *P*=.02; relative risk ratio, 0.709 [95% CI 0.519-0.969]).

**Table 2:** Primary and secondary outcomes assessed at the 4-month checkups (N=265).

Outcomes			Intervention (n=138)		Control (n=127)		Difference (95% CI)		*P* value
Atopic dermatitis, n (%)			28 (20)		42 (33)		–0.13 (–0.23 to –0.02)		.02
**Parenting stress and mental status of mothers**									
	PSI-SF^a^ (total stress), mean (SD)	39.1 (9.8)		37.1 (9.1)		1.95 (–0.34 to 4.25)		.10	
	PSI-SF (child domain), mean (SD)	18.2 (5.4)		17.3 (5.0)		0.89 (–0.36 to 2.14)		.20	
	PSI-SF (parent domain), mean (SD)	20.9 (5.7)		19.8 (5.3)		1.07 (–0.27 to 2.41)		.18	
	GHQ-12^b^≧4, n (%)	45 (33)		44 (35)		0.02 (–0.09 to 0.13)		.79	
**Developmental quotient**									
	Locomotion, mean (SD)	100 (28)		102 (28)		–1.97 (–8.79 to 4.84)		.37	
	Hand exercise, mean (SD)	112 (24)		105 (28)		6.92 (0.52 to 13.33)		.08	
	Common practice, mean (SD)	65 (21)		65 (21)		0.43 (–4.57 to 5.43)		.87	
	Human interaction, mean (SD)	118 (21)		116 (23)		2.22 (–3.05 to 7.48)		.61	
	Speech, mean (SD)	110 (26)		105 (25)		5.10 (–1.16 to 11.36)		.11	
	Language comprehension, mean (SD)	55 (41)		54 (39)		1.18 (–8.53 to 10.88)		.99	
	Average^c^	93 (16)		91 (18)		2.31 (–1.87 to 6.49)		.28	
**Breastfeeding rate**									
	100%, n (%)	79 (57)		59 (46)		0.11 (–0.01 to 0.23)		.09	
	≧80%, n (%)	104 (75)		95 (75)		0.01 (–0.10 to 0.11)		.99	
	0%, n (%)	8 (6)		9 (7)		–0.01 (–0.07 to 0.05)		.80	

^a^PSI-SF: Parenting Stress Index-Short Form.

^b^GHQ: General Health Questionnaire.

^c^This value represents the average of the 6 subscores of the developmental quotient.

## Discussion

### Prevalence of AD in Infants

This study found that the teleconsultation service targeting mothers significantly reduced the prevalence of AD among infants in the intervention group relative to those in the control group. The findings highlight the significance of eHealth and teleconsultation service as an effective management strategy for pediatric AD. Preventing skin problems among infants enhances their quality of life and mitigates against the future development of other allergic diseases [43,44]. Infants with an early eczema onset, especially within 4 months after birth, are at an increased risk of developing food allergies at 3 years of age [45]. Moreover, AD is also known to decrease quality of life among family members and caregivers, deteriorating personal relationships, psychosocial functioning, sleep, and finances [46-48]; the parents’ ability to manage their children’s behavior and illness is influenced by the severity of AD [49]. Our findings show that interventions that facilitate parents’ active engagement can improve treatment outcomes and the holistic well-being of the family.

The intention-to-treat analysis showed that the prevalence of AD based on the United Kingdom Working Party criteria was significantly lower in the intervention group than in the control group at the 4-month checkup. This result suggests that combining typical pediatric services with teleconsultation and email newsletters is effective in preventing AD in infants. We attribute the reduced prevalence of AD in the intervention group to several possible reasons. One reason may be that Syounika Online recommends daily moisturizing skin care based on the reports that this can reduce the risk of AD or eczema and improve skin barrier function [42,50]. Participants who adhered to the service’s advice may have prevented the onset of AD. Another reason may be that frequent email newsletters with advice on skin care for infants might increase parents’ awareness about the importance of infant skin problems or trigger them to visit the hospital earlier than when they otherwise might.

The per-protocol analysis comparing the 3 groups showed a significant difference in the prevalence of AD among them, which suggests that the combined service with teleconsultation and email newsletters and the service with email newsletters alone could be effective in reducing the prevalence of AD. This finding is supported by Santer et al, who reported that a web-based intervention to support families of children with AD had the potential to improve Patient-Oriented Eczema Measure scores [18].

If we consider that fewer participants in the intervention group dropped out than in the control group, then assuming that the 2 dropouts in the former developed AD and the 11 dropouts in the latter did not, the prevalence of AD would be 21% in the intervention group and 30% in the control group. This does not seem inconsistent with the original result, although the difference in the dropout rates is a limitation of this study.

### Parenting Stress and Mental Status of Mothers

The PSI-SF scores observed in the groups of our study were similar to those in a normal population, as indicated by a previous study that reported mean PSI-SF scores of 17.0 in the child domain and 19.4 in the parent domain [51] at 4 months. In contrast, the prevalence of high GHQ-12 scores was over 30% in the intervention and control groups, which is substantially higher than the 7.7% that Yamamoto et al found among postpartum Japanese women at 4 months after delivery [40]. The higher level of parental or psychological stress observed in the mothers in our study indicates the need for further interventions to reduce the stress level among this population.

The lack of difference in maternal scores related to parenting stress and mental health status across the intervention and control groups suggests that although the combination of typical pediatric services with teleconsultation and email newsletters may be ineffective for reducing parenting stress, it does not burden mothers with the information delivered via the service. In addition, we could not assess the impact of the telehealth interventions because logistical difficulties prevented us from being able to collect baseline data on maternal mental health. The intervention group may have contained a higher proportion of stressed mothers than the control group despite the randomization procedure.

Second, PSI-SF and GHQ-12 scores might be inappropriate outcome measures for this study. Ngai et al reported that telephone-based cognitive behavioral therapy was associated with significantly lower levels of parenting stress using the Chinese version of the PSI-SF [30]; however, we used the Japanese version of PSI-SF, which consists of a different number of items. Notably, our teleconsultation service did not specifically target maternal mental health, but it rather focused more broadly on pediatric health and parenting. Sawyer et al reported that an app-based nurse-moderated program was not effective in reducing depressive symptoms measured using the Parenting Sense of Competence Scale and did not improve maternal caregiving measured using the PSI [31]. In contrast, Shorey et al reported that a technology-based supportive educational parenting program was effective in enhancing parenting self-efficacy measured using the Parenting Efficacy Scale, parental bonding measured using the Parent-to-Infant Bonding Questionnaire, perceived social support measured using the Perceived Social Support for Parenting, and parenting satisfaction measured using an evaluation subscale of the What Being a Parent of a Baby Is Like in reducing postnatal depression measured using the Edinburgh Postnatal Depression Scale and postnatal anxiety measured using the State-Trait Anxiety Inventory [32]. Future research should have a different perspective on the parenting stress observed in this study and use appropriate measurements such as the Parenting Efficacy Scale, Parent-to-Infant Bonding Questionnaire, Perceived Social Support for Parenting, What Being a Parent of a Baby Is Like, Edinburgh Postnatal Depression Scale, or State-Trait Anxiety Inventory to assess the postpartum mental status of mothers.

### Limitations

There were several limitations in this study. First, more participants in the intervention group than in the control group dropped out, although this may not have had a profound impact on the results according to the imputation. Second, the participants with a family history of AD were less common in the intervention group; however, the subgroup analysis showed that there was no significant difference in the prevalence of AD. Third, because we adopted a combined service with teleconsultation and email newsletters as the intervention, it was not possible to deduce the individual effectiveness of the interventions. Fourth, due to the limited duration of this study, we collected the outcome data only once and could not follow up on the participants for a longer period. Fifth, we could not collect the skin treatment history of the infants and baseline data on maternal mental health. Future studies are required spanning a longer duration at multiple sites to provide evidence for long-term effectiveness and more detailed information.

### Overview

This study examined the effectiveness of a combined pediatric service with teleconsultation and email newsletters among infants and mothers in Japan. As we recruited pairs of infants and mothers from a whole ward and 60% of these recruited pairs were analyzed, our findings should be generalizable to many other areas in Japan whose surroundings are similar. The intervention to support infants and mothers by pediatricians via smartphone-based internet services was novel in Japan. To the best of our knowledge, this is the first RCT to show an association between eHealth and a reduced prevalence of AD in infants.

### Others: DQ of Infants and Breastfeeding Rates

There was no difference in the DQ between the intervention and control groups. The DQ of hand exercise in the intervention group tended to be higher; however, the difference has little clinical relevance. As the DQ is based on developmental and chronological ages, a longitudinal study might provide more accurate information about the long-term effects of interventions on the DQ. Future studies should investigate long-term effectiveness because we assessed infants’ development only at 4 months of age.

Although there was also no difference in breastfeeding rates between the intervention and the control groups, more infants in the intervention group tended to receive exclusive breastfeeding and fewer tended to receive exclusive formula feeding. This distinction may indicate that a combined pediatric service with teleconsultation and email newsletters could encourage mothers to breastfeed. Future research should incorporate larger studies to address this topic.

### Adverse Events

There were no adverse events in this trial.

### Conclusions

Although a combined pediatric service with teleconsultation and email newsletters was not effective in reducing parenting stress in mothers, this is the first RCT to demonstrate that telehealth was effective in reducing the prevalence of AD in infants. Such educational programs are a necessary complement to clinical and public services attempting to improve skin conditions in infants. We hope that pediatric eHealth will become a new, widely used strategy for preventing AD, which may also lead to a reduction in other allergic diseases.

## Appendix

Multimedia Appendix 1 CONSORT-eHEALTH checklist (V 1.6.1).

## Abbreviations

AD: atopic dermatitis
DQ: developmental quotient
GHQ-12: General Health Questionnaire-12
PSI-SF: Parenting Stress Index-Short Form
RCT: randomized controlled trial
SIMPLE: Sakae Infants and Mothers with the Pediatric Teleconsultation
