# Correction: Effectiveness of Pediatric Teleconsultation to Prevent Skin Conditions in Infants and Reduce Parenting Stress in Mothers: Randomized Controlled Trial

**DOI:** 10.2196/38059

**Published:** 2022-04-12

**Authors:** Tomohisa Ando, Rintaro Mori, Kenji Takehara, Mari Asukata, Shuichi Ito, Akira Oka

**Affiliations:** 1 Department of Pediatrics University of Tokyo Tokyo Japan; 2 Department of Health Policy National Center for Child Health and Development Tokyo Japan; 3 Population Ageing and Sustainable Development Asia and the Pacific Regional Office United Nations Population Fund Bangkok Thailand; 4 Tsurumi Ward Administration Office Yokohama Japan; 5 Department of Pediatrics Graduate School of Medicine Yokohama City University Yokohama Japan

In “Effectiveness of Pediatric Teleconsultation to Prevent Skin Conditions in Infants and Reduce Parenting Stress in Mothers: Randomized Controlled Trial” [JMIR Pediatr Parent 2022;5(1):e27615] the authors noted two errors.

First, in the Abstract; Results section of the originally published article the significant difference was reported as follows:

20% vs 33%, P=.03; relative risk ratio, 0.614 [95% CI 0.406-0.927]

This has been corrected as follows:

20% vs 33%, P=.02; relative risk ratio, 0.709 [95% CI 0.519-0.969]

Second, in Table 2 of the originally published article the *P* value for “Atopic dermatitis” was reported as follows:

P=.03

This has been corrected as follows:

P=.02

The correction will appear in the online version of the paper on the JMIR Publications website on April 12, 2022, together with the publication of this correction notice. Because this was made after submission to PubMed, PubMed Central, and other full-text repositories, the corrected article has also been resubmitted to those repositories.

